# Data Management and Modeling in Plant Biology

**DOI:** 10.3389/fpls.2021.717958

**Published:** 2021-09-03

**Authors:** Maria Krantz, David Zimmer, Stephan O. Adler, Anastasia Kitashova, Edda Klipp, Timo Mühlhaus, Thomas Nägele

**Affiliations:** ^1^Theoretical Biophysics, Institute of Biology, Humboldt-Universität zu Berlin, Berlin, Germany; ^2^Computational Systems Biology, Technische Universität Kaiserslautern, Kaiserslautern, Germany; ^3^Plant Evolutionary Cell Biology, Faculty of Biology, Ludwig-Maximilians-Universität München, Planegg-Martinsried, Germany

**Keywords:** genome-scale networks, *omics* analysis, metabolic regulation, plant-environment interactions, machine learning, mathematical modeling, differential equations

## Abstract

The study of plant-environment interactions is a multidisciplinary research field. With the emergence of quantitative large-scale and high-throughput techniques, amount and dimensionality of experimental data have strongly increased. Appropriate strategies for data storage, management, and evaluation are needed to make efficient use of experimental findings. Computational approaches of data mining are essential for deriving statistical trends and signatures contained in data matrices. Although, current biology is challenged by high data dimensionality in general, this is particularly true for plant biology. Plants as sessile organisms have to cope with environmental fluctuations. This typically results in strong dynamics of metabolite and protein concentrations which are often challenging to quantify. Summarizing experimental output results in complex data arrays, which need computational statistics and numerical methods for building quantitative models. Experimental findings need to be combined by computational models to gain a mechanistic understanding of plant metabolism. For this, bioinformatics and mathematics need to be combined with experimental setups in physiology, biochemistry, and molecular biology. This review presents and discusses concepts at the interface of experiment and computation, which are likely to shape current and future plant biology. Finally, this interface is discussed with regard to its capabilities and limitations to develop a quantitative model of plant-environment interactions.

## Introduction

Experimental high-throughput analysis of genomes, transcriptomes, proteomes, and metabolomes results in a vast number of simultaneously quantified molecular entities. Current biological research frequently applies a combination of experimental high-throughput techniques to address a wide spectrum of complex research questions. On the genome level, high-throughput sequencing (HTS) technologies have revolutionized genetics and genomics, and sequencing projects have provided comprehensive information about many species’ genomes (Lander et al., 2001; The 1000 Genomes Project Consortium, 2012; The 1001 Genomes Consortium, 2016; Stein et al., 2018; Sun et al., 2019). To date, thousands of genomes have been sequenced and pan-genomics approaches have been initiated which assemble diverse sets of individual genomes to a collection of all DNA sequences occurring in a species (Sherman and Salzberg, 2020). In plant sciences, the concept of pan-genomics is already discussed to support breeding strategies or evolutionary studies and may significantly contribute to the explanation of gene presence and absence variation (Bayer et al., 2020).

Based on such comprehensive genome information, genome-scale models of plant metabolism have been developed and applied to predict plant metabolism in a diverse context. Validation and biotechnological application of such large-scale models need appropriate experimental techniques and platforms, unifying sample analysis in multi-*omics* approaches (Weckwerth et al., 2020). Although, *omics* techniques have become a generic element of numerous research projects to quantify transcripts, proteins and metabolites, handling, normalization, and integration of the multidimensional experimental data output is still a central challenge in biology (Scossa et al., 2021). The need for integrative analysis of experimental high-throughput data has already been suggested and discussed earlier. For example, almost a decade ago, integrative approaches were suggested for transcriptomics, proteomics, and metabolomics data to promote a systems-level understanding of *Arabidopsis* (Liberman et al., 2012). Since then, machine learning, computational statistics and mathematical modeling have significantly advanced data integration strategies. Due to their capability to improve the understanding of the genotype-phenotype relation on a molecular level, systems biology, and multi-*omics* integration have become central topics in the discussion about future perspectives of biology and medicine. Yet, in order to make experiments comparable and to increase consistency and reproducibility across different experimental platforms, laboratories, or research communities, quantitative *omics* data are needed (Pinu et al., 2019). Furthermore, quantitative experimental data necessitates appropriate processing strategies to make it comparable to other independent studies and statistics. Making data and data processing publicly available *via* databases and repositories may represent one of the most important steps to establish and expand a cross-disciplinary scientific platform for *omics* data integration. Together with the need for traceable long-term data storage and versioning, these topics are becoming increasingly important in quantitative biology.

**Figure 1:**
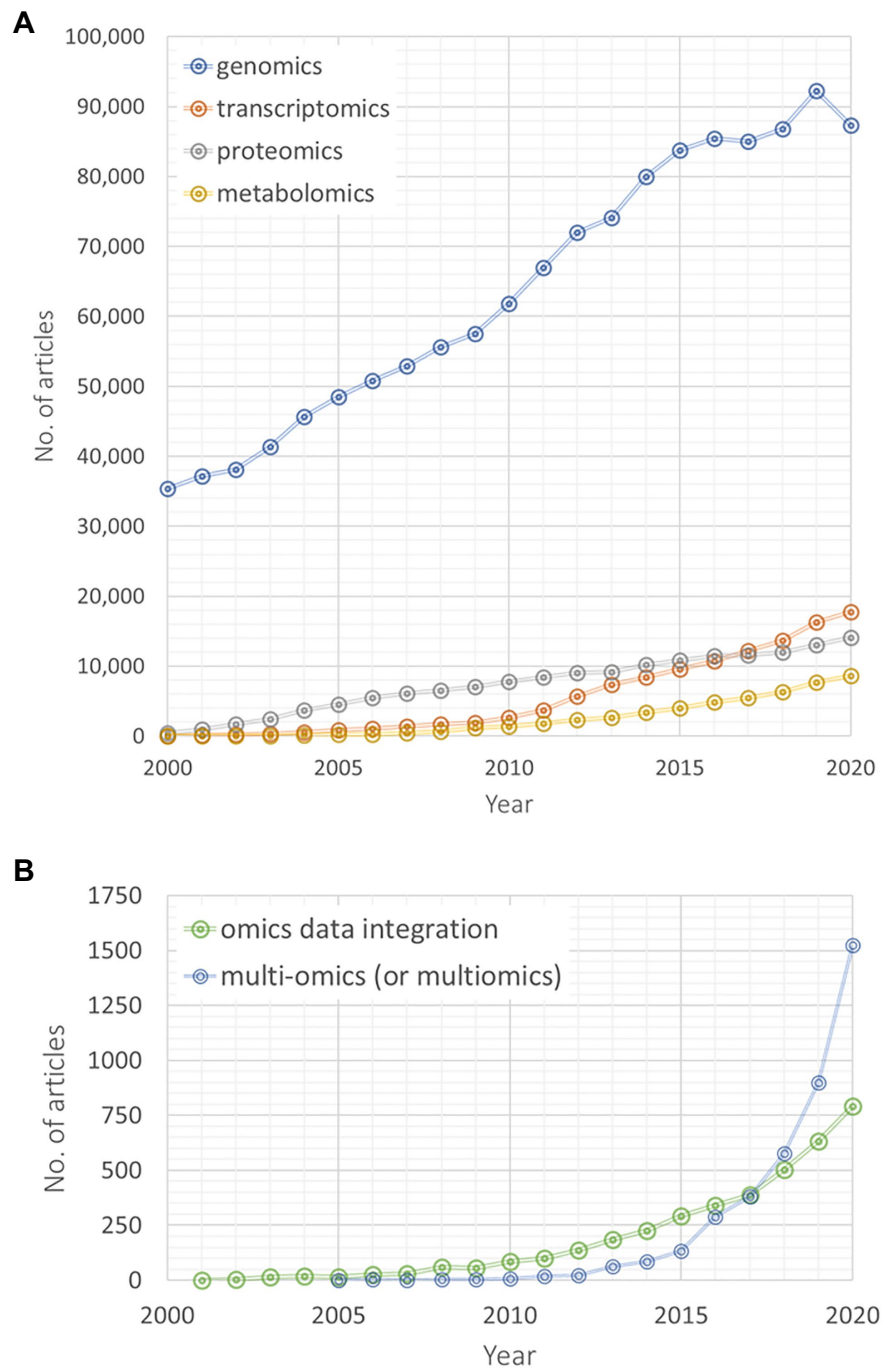
Number of articles found by article search in the PubMed® library covering 2 decades, i.e., 2000–2020 (https://pubmed.ncbi.nlm.nih.gov). **(A)** Timeline of number of articles on different *omics* disciplines (blue: genomics; orange: transcriptomics; gray: proteomics; and yellow: metabolomics). Articles were searched by single key word search, **(B)** Timeline of number of articles found by search on *omics* data integration (green line; single words were connected by AND-expression) and multi-*omics* (or multiomics, blue line).

Searching for data base entries of the last 2 decades on *omics* and integrative *omics* approaches reveals a rapidly increasing research and publication activity in the integrative multi-*omics* research field (Figure 1; results from a search performed on PubMed®).^1^ Genomics-related yearly published articles linearly increased on a very high level during the last 20years, while particularly transcriptomics and metabolomics articles are published with an increasing rate during the last decade (Figure 1A). Between 2000 and 2015, more proteomics-related articles have been published than transcriptomics and metabolomics articles but since 2017 their number lies between both *omics* disciplines. Interestingly, since 2017, articles searchable by queries “multi-*omics*” (or “multiomics”) are exponentially increasing in their number (Figure 1B). A similar, yet weaker trend is also observable for “*omics* data integration” articles (Figure 1B). Of course, these numbers are only crude estimates based on our chosen specific vocabulary and searched within one specific database (for example, we have not checked the combination of different *omics* disciplines, i.e., “genomics” and “transcriptomics” instead of “multi-*omics*”). Yet, these results still indicate that an increasing number of studies focuses on a multi-*omics* design and that *omics* data integration gains more and more attention. This article aims to summarize and discuss current advances and limitations of integrative molecular analysis, computational modeling, and data science. It focuses on both experimental and theoretical methodology to support design and analysis of interdisciplinary research in plant biology. A particular focus is laid on methodologies for capturing system dynamics of plant metabolism induced by a changing environment.

## On a Large Scale: How Does Genome-Scale Metabolic Network Reconstruction Support Data Integration in Plant Biology?

The availability of comprehensive genome information has enabled the reconstruction of genome-scale metabolic networks, which predict, based on gene annotation, a functional cellular network structure. This crucially supports the interpretation of gene functions and makes pathways accessible to computational biology and mathematics (Oberhardt et al., 2009). Further, reconstructed networks significantly facilitate a mechanistic description of genotype-phenotype relationships and enable the application of constraint-based analysis methods (Lewis et al., 2012; Ramon et al., 2018). Major constraints are thermodynamics, mass and charge conservation and the substrate/enzyme availability. Constraints dramatically reduce the parameter space, which explains a genotype-phenotype relationship, and, hence, strongly increases the probability to find physiologically relevant solutions for underlying equation systems. Thus, it is not surprising that, in current plant biology, genome-scale reconstruction has become an integral part from single-cell to multi tissue modeling (Gomes de Oliveira Dal’Molin and Nielsen, 2018). For example, model reconstructions have been applied to analyze metabolic regulation in autotrophic and heterotrophic tissues, to study C4 plant metabolism, to evaluate diurnal metabolic interactions in plant leaf tissue and to analyze photorespiration (de Oliveira Dal’Molin et al., 2010a,b; Cheung et al., 2014; Yuan et al., 2016).

The experimental basis for constraining, validating, and optimizing large-scale models are high-throughput experiments, i.e., *omics* analyses. For example, to investigate effects of nitrogen assimilation on metabolism in maize (*Zea mays*), a genome-scale metabolic model for maize leaf was created comprising more than 5,800 genes, 8,500 reactions, and 9,000 metabolites (Simons et al., 2014). Using a combination of transcriptomic and proteomic data to constrain metabolic flux predictions, the authors were able to reproduce experimentally determined metabolomic data to significantly higher accuracy than without these constraints. Applying a combination of publicly available data on maize metabolism, reaction networks, and results from *omics* experiments, information about reaction stoichiometry, directionality, and compartmentalization was derived. Algorithmic model curation was combined with manual modification to, for example, resolve gaps in the network model with reactions from similar organisms. Information about transcripts and proteins, which were experimentally observed to significantly differ in mutants and under variable nitrogen supply, were then incorporated into the model by switching on/off corresponding reactions. Flux predictions through the metabolic network were compared to metabolomics measurements. With this integrated setup, model application unraveled genes coding for enzymes, which are involved in regulation of biomass formation under variable nitrogen supply (Simons et al., 2014). In another study, publicly available transcriptomics and metabolomics data were used within a constraint-based modeling approach to investigate network structure and flux distribution in root cell types and tissue layers of *Arabidopsis thaliana* (Scheunemann et al., 2018). Based on transcriptomics and metabolomics data, it was possible to extract tissue and cell type specific models from a general genome-scale model of root metabolism. By this, the authors were able to simulate and analyze cell types as autonomous subsystems, which communicate with each other *via* metabolites or proteins. But it was also shown and discussed that further experimental evidence and constraints are essential to support hypotheses derived from their simulations (Scheunemann et al., 2018). This example nicely illustrates how large-scale data integration can (i) unravel novel and detailed mechanistic insights into plant metabolism, and also (ii) indicate design and research focus of follow-up studies to prove model predictions. By placing metabolites, proteins, or transcripts into a pathway and network context, genome-scale models significantly support the biochemical and physiological interpretation of molecular data.

Also, in a biotechnological context, such data integration strategies have become an important and promising tool to advance and improve bioengineering strategies. As an example, a genome-scale metabolic network reconstruction for green microalgal model species *Chlamydomonas reinhardtii* has been developed which reliably and quantitatively predicted growth depending on the light source (Chang et al., 2011). This metabolic network comprises 10 compartments, accounts for more than 1,000 genes associated with more than 2,000 reactions and over 1,000 metabolites. Regulatory effects arising from different light conditions are covered by the model, which enables estimation of growth under different laboratory conditions. The model has been refined using metabolite profiling to include further branches of metabolism, e.g., amino acids and peptides as nitrogen sources (Chaiboonchoe et al., 2014). Although, it has been developed a decade ago, the original model (named iRC1080) still represents a valid and supportive platform for data interpretation, and it still fruitfully initiates further model development and validation, see e.g., Shene et al. (2018). These examples, together with many other studies which were summarized recently (Tong et al., 2021), provide strong evidence for the capability of genome-scale metabolic models to couple statistics with metabolic models.

## Large-Scale Models Need Quantitative Large-Scale Experiments on Integrative Platforms for Validation and Iterative Parameter Optimization

Reconstruction of genome-scale metabolic network from genome sequence information is an iterative process, which needs several rounds of automatized and manual model adjustment, reconfiguration and fine-tuning (Thiele and Palsson, 2010). It strictly depends on genome annotation, and due to the strong increase of genome sequence information high-throughput annotation algorithms are necessary to cope with this vast amount of data. Particularly in eukaryotic genomes, annotation errors due to assembly errors are still a challenge in the field, and direct RNA sequencing is discussed to improve gene annotation in the future (for details please refer to Salzberg, 2019; Workman et al., 2019). However, as soon as a model has been curated and applied to predict metabolic flux or growth, quantitative experiments are needed to validate the model output, and to iteratively adjust model parameters. In addition to validation variables like growth rates, lipid content, ATP concentration, or total protein amount, experimental *omics* analyses potentially provide detailed information about pathway regulation, gene regulatory networks and signaling cascades. Here, mass spectrometry-based proteomics and metabolomics analyses play a crucial role which are not only able to analyze posttranslational modifications or protein localization, but also can quantify turnover rates and metabolic fluxes down to subcellular scale (Szecowka et al., 2013; Chen et al., 2021).

Quality of experimental data limits optimization of *in silico* models. If absolute quantitative model predictions about metabolite or protein dynamics cannot be experimentally validated due to missing absolute quantitative experiments, accuracy, and reliability of the model frequently remain ambiguous or elusive. Several complex and non-intuitive questions about stability or regulatory patterns might still be addressed with such a model. Yet, the physiological constitution of a plant, or organism in general, which results from a certain growth setup, can hardly be modeled and simulated without quantitative information. For example, plant growth strictly depends on various growth parameters, e.g., light intensity and quality, soil composition, water availability, and humidity. It is well known that a slight modification of only one of those growth parameters might strongly affect the (molecular) phenotype which makes comparative studies difficult. For example, different light sources might be applied (LEDs, fluorescent tubes, etc.) in different laboratories, which immediately results in different growth behavior and physiological properties (Seiler et al., 2017). While global harmonization of growth cabinets, greenhouses, or climate chambers remains impractical, augmentation of quantitative *omics* analysis seems realistic. Recommendations and potential pitfalls of experimental designs are already discussed on a research community level (Pinu et al., 2019). The authors recommend quality control samples (QCs) and universal standardized operating protocols (SOPs) for quantitative and reproducible experiments. Further, collecting and publishing comprehensive meta-data is recommended to guide through and inform about experiments (Ara et al., 2015; Meyer, 2015; Kale et al., 2016).

In plant biology, absolute quantification of primary and secondary metabolites might represent a suitable approach to make studies comparable across platforms and growth regimes. Plant metabolism shows a high plasticity across different diurnal light periods, e.g., under short day growth conditions with 8h light and 16h darkness, dynamics of sugar and amino acid concentrations are significantly stronger than under long day growth conditions, i.e., under 16h light and 8h darkness (see e.g., Sulpice et al., 2014). Additionally, the ratio of monosaccharides and disaccharides may vary significantly between growth setups, which is not detectable within a qualitative *omics* study because it does not allow the absolute comparison of two or more different substances. In mass spectrometry, one reason for this is that different molecules, e.g., sucrose and glucose, produce different ions with different masses, which are detected with different intensity. Hence, to make resulting mass spectra and chromatographic peaks comparable across different substances, they need to be individually scaled by a dilution of standard substances, i.e., within a calibration curve, yielding absolute amount of substance within a sample, which can then be normalized to sample protein amount or sample weight. Depending on the applied growth conditions and treatment, normalization might either be favorable to fresh or dry weight. For example, exposing plants to heat and/or drought stress directly affects leaf water content and, thus, under such conditions normalization to dry weight should be favored if metabolite concentrations are quantified.

While such an approach is appropriate for absolute quantification of central primary metabolites, i.e., sugars, amino acids, or organic acids (Weiszmann et al., 2018), it is hardly feasible for each individual substance within a metabolite profile. For many substances, appropriate standard substances are lacking, and even if they are available, they might be expensive due to costly purification and/or synthesis procedures. Further problems might occur when purified substances, like polar and apolar amino acids, need to be diluted and mixed within calibration samples due to their different solubilities in water. The vast number of metabolites, which are estimated to comprise between 200,000 and 1 million across the plant kingdom and up to 5,000 within a single species (Fernie et al., 2004; Fang et al., 2019), makes quantitative metabolomics challenging. Based on these numbers, it seems unfeasible to resolve quantity of hundreds or thousands of compounds within a GC-MS or LC-MS run. While combination of different analytical platforms promises to cover a large panel of compounds (Pazhamala et al., 2021; Zancarini et al., 2021), semi-quantitative analysis might represent a suitable approach to increase reproducibility and comparability of high-throughput analysis among quantification platforms. Here, structural elucidation of metabolic compounds based on mass spectrometry data might indicate a compound’s class (De Vijlder et al., 2018). This information, together with chromatographic information about retention time or index, might allow classification of an unknown substance by data base search and comparison to known substances with similar mass spectra and physical properties like polarity. This would enable the comparison of chromatographic peak areas of an unknown substance to a known and most similar standard substance. For example, an unknown substance which, based on its mass spectrum information, is predicted to be a disaccharide might be semi-quantified applying the calibration of a known disaccharide with similar retention time or index. In this way, semiquantitative information of an unknown substance might be derived from GC-MS (primary metabolites) or LC-MS (secondary metabolites) run which would facilitate comparison and data exchange of independent studies and on different experimental platforms.

## Research Data Management Provides the Groundwork for Successful Data Integration

Data integration methods, especially machine learning approaches, profit heavily from the increasing availability of data. Aside from high-dimensionality and sparsity of biological data, a fundamental challenge in data integration lies in accessibility and quality of information and knowledge. Modern approaches require not only massive, but particularly well-annotated data sets (Webb, 2018).

Currently, the default medium of scientific communication in the domain of biology is the publication in peer reviewed scientific journals centered around free text-based communication. While this format has many benefits such as the quality control by curators being experts on the respective field, it also has the drawback of being gated by pay walls. This issue is already being addressed with the increased founding of open access journals, but the approach suffers from more intrinsic problems. The format itself was designed as a human readable medium and is thus prone to design flaws that can be implicitly solved by a human reader but imposes problems to the application of machine learning techniques. Examples being the heterogeneity of supplementals, the embedding of data as schematic descriptions, and most severely, the communication of findings as free text. While these challenges are already identified and currently tackled by manual curation and the application of natural language processing (NLP) and pattern recognition, its frequent occurrence still hinders the direct computational usage of the published knowledge for data integration (Karp, 2016).

An alternative approach of scientific communication is realized by the creation of knowledge databases. In plant research, there are various information resources and data portals of extremely high quality. UniProt (UniProt Consortium, 2019) and Ensembl plants (Bolser et al., 2016) are integrative resources presenting genome-scale information for a growing number of sequenced plant species. Additionally, PLAZA (Van Bel et al., 2018) provides an integrative resource for functional, evolutionary, and comparative plant genomics. Data portals and specific databases like the “The Arabidopsis Information Resource” (TAIR; Berardini et al., 2015), Araport (Krishnakumar et al., 2015), Aramemnon (Schwacke et al., 2003), or Phytozome (Goodstein et al., 2012), provide fine-grained species-specific reference knowledge. Generally, these resources offer a more condensed compilation of knowledge and often preserve the virtue of being manually curated. However, each iteration of a knowledge database only represents a snapshot of the knowledge at the time of creation, which imposes the initiator with the additional burden of maintenance and the user with uncertainty with regards to the currentness of the data source. In comparison to free text, knowledge data bases are often easier to access by computational means and provide a better interoperability when it comes to the application of ML methods, nevertheless they were and still are designed with a human operator in mind and often lack important meta data information. This does not only affect processes like data retrieval but also the documentation of how data was obtained and integrated when assembling the database.

The communication of findings in scientific publications or their integration in knowledge data bases is of course limited by the questions asked at the time of creation. Therefore, best practice suggests publishing raw measurements data in a technology-specific data repository. ProteomeXchange (Vizcaíno et al., 2014), Gene Expression Omnibus (GEO; Clough and Barrett, 2016), SRA/ENA (Leinonen et al., 2010), and Metabolights (Haug et al., 2013) are well established data exchange platforms that enforce certain metadata annotation tailored to the individual technology. Generic data repositories like figshare^2^ and Dataverse^3^ do not require a technology-specific and laborious annotation process, but in turn do not ensure the necessary metadata annotation. Repositories can improve the process of peer review since the evaluation of data itself can be analyzed with respect to their reproducibility and also make the raw data accessible to the community for reevaluation. This allows to test new hypothesis using existing data sets. Nonetheless, the reuse of published data sets is limited by the level of detail in which their creation is described. Therefore, consortia and initiatives coordinate standardization efforts in plant research and developed standards and checklists to formally enable researchers to communicate their findings with required meta data. In the plant field, excellent standardizations for experimental data collections are the “Minimal Information on Biological and Biomedical Investigations” (Taylor et al., 2008), “Minimal Information about a Plant Microarray Experiment” (Zimmermann et al., 2006), and “Minimal Information about Plant Phenotyping Experiments” (Krajewski et al., 2015). However, it is exceedingly difficult for researchers to judge the necessity of certain meta information beforehand. Additionally, considerable effort and skills are required to provide adequate metadata annotation to the research data. Researchers also need to allocate the resources and capacity to actually do so in daily research practice. In addition, many researchers view data as sensitive research output that could easily be misused or mis-interpreted when taken out of context. Thus, many scientists do not trust global repositories unless they have direct and personal connections to these researchers’ own work or find it too time consuming to validate their trustworthiness.

Nevertheless, it is evident that all ways of research communication e.g., scientific journals, knowledge databases, and data repositories, heavily benefit from improved meta data description, not only in terms of reproducibility, but also accessibility and thus reusability (Leonelli, 2019). It is apparent that research data management requires a constant endeavor of researchers and well accepted standards need to be developed. Here, the FAIR principles form a conceptual roof and formulate the necessary goals to achieve (Wilkinson et al., 2016). The FAIR data principle is founded on four core elements: (i) findability, (ii) accessibility, (iii) interoperability, and (iv) re-usability. Findable data is described/annotated with rich metadata and consists of a globally unique identifier, which is indexed in a searchable source, e.g., a database. The metadata must specify what kind of identifier is used. According to the accessibility/accessible, metadata and data must be retrievable based on their identifier by using a standardized protocol, which is open and universally implementable. Interoperable data use a standard vocabulary based on the FAIR principles and include qualified references to other (meta)data and most importantly are represented using a formal, accessible, shared, and broadly applicable language for knowledge representation. Consequently, re-usable (meta)data have a plurality of accurate and relevant attributes. In addition, they need to be associated with their provenance and meet domain specific community standards.

Generic implementations to assist researcher to abide by the FAIR principles have already been implemented. The usage of Research Object (Hettne et al., 2014), Research Object Crate (RO; Carragáin et al., 2019), or ISA data model (Gonzalez-Beltran et al., 2014) can lead to a rich description of the experimental metadata (i.e., sample characteristics, technology and measurement types, sample-to-data relationships) that make the resulting data and discoveries reproducible and reusable. Scientific findings accompanied with rich meta data descriptions are representable as knowledge graphs. Such graphs greatly improve their value to the scientific community, since the embedding into traversable tree-like structures results in a cross linking of available scientific data, which makes knowledge searchable. In practice this is achieved using domain specific ontologies, which constrain the used vocabulary as well as conserving the relationship of single terms.

Reproducibility and provenance play an important role especially in the computational analysis itself. Recent efforts to make analytic pipelines independent of their runtime environment strongly improved reusability and reproducibility of workflows. Containerization of processing tools and analytic pipelines facilitate the sharing and collaborative development of workflows on specialized platforms like WorkflowHUB. Analogously, computation requires meta data and specifications. In this regard, the BioCompute Object Project (Simonyan et al., 2017) aims to ease the exchange of HTS workflows between various organizations by providing a json format that, at a minimum, contains all the software versions and parameters necessary to evaluate or verify a computational pipeline.

It becomes evident that a combination of computation, data and their meta data is essential to achieve the common goal of a well annotated research object living up to the FAIR principles (Simonyan et al., 2017; Vicente-Saez and Martinez-Fuentes, 2018). Therefore, community driven initiatives like DataPLANT support plant scientists in every research data management concern and provide a tailor-made service environment to contextualize research data according to the FAIR principles with minimal additional effort in modern plant biology.

## Recognizing Patterns and Quantifying Dynamics of Plant Metabolism: Where Biology Meets Mathematics and Informatics

### Machine Learning and Its Role in Quantitative Plant Biology

The rapid development of experimental high-throughput techniques together with a significant drop of costs per sample have made *omics* analyses become a common element of experimental biology (Weckwerth et al., 2020). Resulting data matrices are high dimensional and, thus, reduction of dimensions to those which explain most of observed variance within a sample set is a routinely applied method. Principal component analysis (PCA) represents a method of unsupervised learning, and, in more detail, it belongs to a branch called *distributed representation* (Skansi, 2018). Another branch of unsupervised learning, which is frequently applied in biology, is *clustering*. Together with supervised learning and reinforcement learning, unsupervised learning represents a main subdiscipline of machine learning (Skansi, 2018). Machine learning itself is a subfield of artificial intelligence (AI), which has gained rapidly increasing attention across many biological disciplines during the last decade (Li et al., 2018; Sun et al., 2020). Supervised and unsupervised learning are two categories of machine learning which differ in the availability, i.e., knowledge, of a response value. In supervised learning, each observation of predictor measurements x_i_ (with observations *i*=1, …, *n*), is associated to a response measurement y_i_. The aim is to fit a model which predicts responses for future observations or which supports the interpretation of predictor-response relationship. In unsupervised learning, predictor measurements x_i_ are available but no response measurements y_i_. Clustering, as an example for unsupervised learning, aims to figure out whether observations can be separated into distinct groups and, by this, understand the relationship between variables. Reinforcement learning aims to “teach” an agent how to interact with the environment to obtain a “good” score under certain preliminary settings (Dong et al., 2020). It plays an important role in many fields of biology, robotics, and health care (Shteingart and Loewenstein, 2014; Esteva et al., 2019) and also gains attention in the field of metabolic engineering. Recently, a reinforcement learning method has been applied for bioretrosynthesis, i.e., the synthesis of organic chemicals from low-cost precursors and enzymes (Koch et al., 2020).

**Figure 2:**
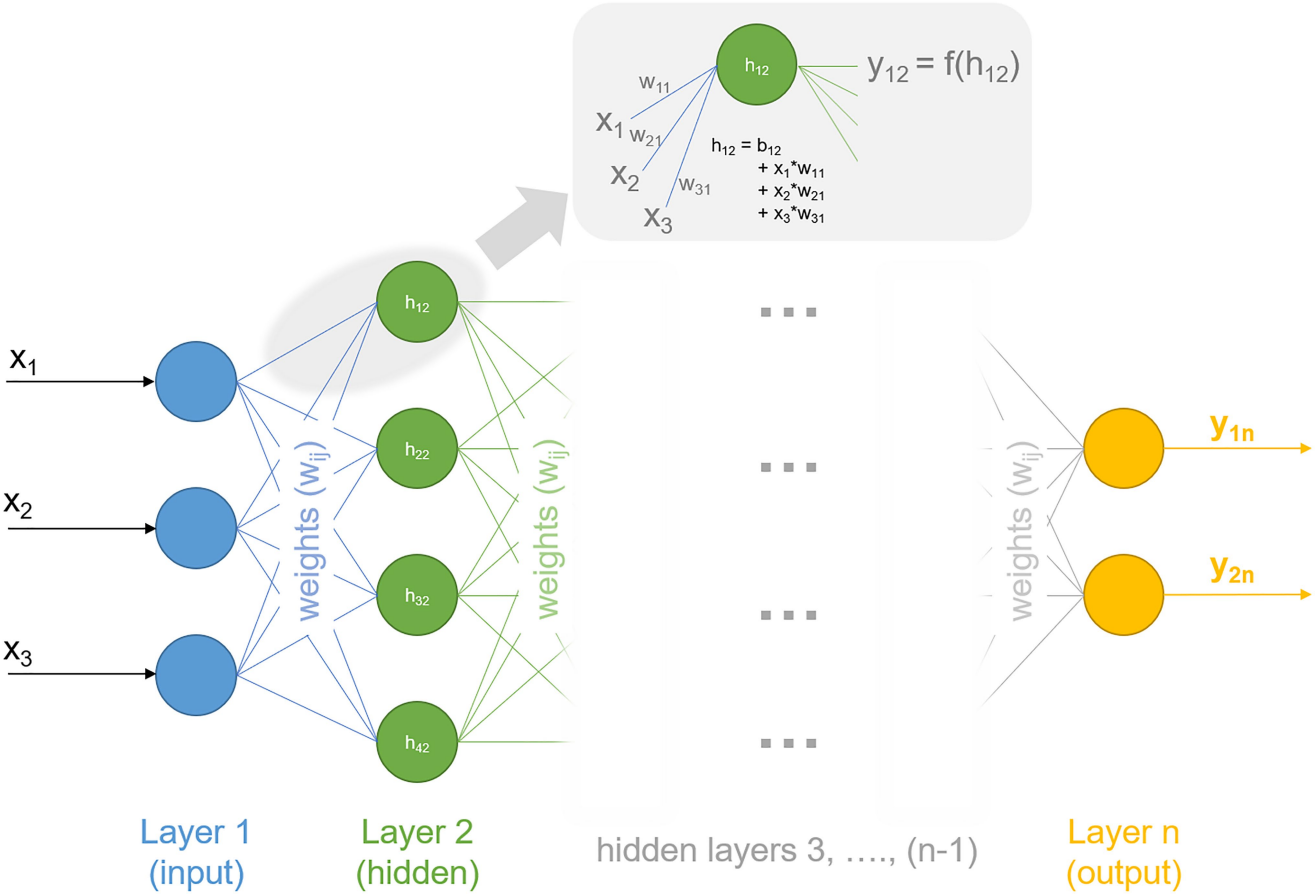
An artificial neural network. Information, i.e., x_1_, x_2_, and x_3_, enters the network *via* the input layer (layer 1, blue). Weights w_ij_ determine the quantity by which information is passed to layer 2 (hidden, green). Processed information, here y_1n_ and y_2n_, leaves the network by the output layer (layer n, yellow). Indices refer to neuron number and layer number, respectively. Calculations for neurons are depicted exemplarily for h_12_ (first neuron in second layer), which is composed of a bias (b_12_) and summed information of the previous layer, here layer 1. Resulting information, y_12_, is passed to the next layer and might comprise nonlinearities in f(h_12_). Deep neural networks typically comprise several and up to numerous hidden layers, indicated in grey.

Deep learning is a family of machine learning methods that comprises algorithms of multi-layered artificial neural networks, i.e., in networks of interconnected neurons which are organized within layers. In principle, deep learning approaches also can be subdivided in previously mentioned supervised, unsupervised, and reinforcement learning and extend them to a category in which a model directly learns from a very large data set. Particularly for massive data sets, deep learning performs better than other machine learning approaches (Bhattacharyya et al., 2020). Recently, protein structure prediction has been considerably advanced by the deep learning system AlphaFold, developed by the Google AI offshoot DeepMind (Senior et al., 2020). AlphaFold applies deep learning to predict backbone torsion angles and pairwise distances between amino acids within a protein based on sequence information and multiple sequence alignment. In deep learning, *deep* refers to the number of layers of neurons – the more layers, the *deeper* the network. The flow of information within a neural network starts from neurons within the first layer, the so-called input-layer. Within a fully connected neural network, each neuron of the input-layer is connected to all neurons of the second layer, every second-layer neuron to each third-layer neuron and so on. Each connection is weighted to determine the quantitative extent to which they are transmitted to the next layer. In addition to weights, each neuron can be modified by a value called *bias*, which is added to the sum of the previous layer. Hence, applying an (artificial) neural network in data analysis means to search for optimal sets of weights and biases to reduce the error between model output and experimental observation and to maximize probability of true predictions. Typically, nonlinearities (or activation functions) are introduced into the network to describe a transfer function f between layers. For example, nonlinearity converts the input signal of layer 1 into an output signal, which represents the input signal for layer 2 (Figure 2). Weights of the network determine the quantity by which information is passed from layer to layer until the processed information leaves the network by the output layer.

This finally represents one central reason why machine learning, and particularly deep learning, are promising and successful strategies for complex, i.e., nonlinear, data analysis: nonlinear functions are employed in different layers to calculate the probability of observing an output due to given input. Examples for such nonlinearities are sigmoid functions and hyperbolic tangent functions (Bhattacharyya et al., 2020). Further, principles like backpropagation are employed for weights learning within an (artificial) neural network, which aim at minimizing error between prediction and observation (for more details see Skansi, 2018). Training data sets are applied for the learning process before the network is applied to the test data set, which has not been seen by the model before. This training/test-set validation provides immediate information about the network performance, which is measured by metrics like *R-squared* for regression problems and the *area under the Receiver Operating Characteristic (ROC) curve* (*auROC*) for classification problems. Also, architecture of neural networks may vary significantly and can, e.g., be discriminated by their number of layers (single or multiple layer width), feedforward (no feedback-loop), or recurrent networks (with at least one feedback-loop within or between layers). The parameter space of neural networks is classified according to their number of layers, the number of neurons within input, hidden and output layers, initial values for weights, initial values for biases, and the occurrence of feedback-loops.

Deep learning has recently gained much attention in the field of plant genomics, proteomics (Zimmer et al., 2018), and crop improvement (for overview articles about current applications refer to, e.g., Wang et al., 2020; Tong and Nikoloski, 2021). Deep learning models are discussed in context of the new breeding era, Breeding 4.0, which largely depends on genome editing and which would significantly benefit from predictions of allele effects (Wang et al., 2020). Predicting how allele effects impact crop yield and general performance under changing environmental conditions would facilitate the identification of molecular traits, which are central for efficient and biomarker-assisted breeding (Wang et al., 2020). Although still being very ambitious, with deep learning such predictions become more likely due to the capability of nonlinear data analysis.

In addition to (applied) crop science, machine learning approaches will crucially support basic plant sciences. Particularly, quantitative analysis of nonlinear plant-environment interactions, which essentially shape plant stress response, acclimation, and adaptation, is raised to the next level of complexity (Khaki and Wang, 2019). Recent work has indicated how machine learning can be employed to predict plant growth based on reaction rates, which were gained from metabolic models (Tong et al., 2020). This shows that machine learning is capable of integrating comprehensive information on different layers of molecular and physiological information. However, it simultaneously emphasizes the need for standardized quantitative high-throughput data for training and testing of machine learning approaches in plant biology (Xu and Jackson, 2019). Plant metabolism remains highly complex and machine learning comprises many mathematical functions, which are hardly interpretable with regard to physiology. This might, in some scenarios, even complicate the validation and interpretation of a machine learning-driven prediction because causal inference of molecular processes is prevented by high algorithmic complexity. Furthermore, estimation of performance and accuracy of deep learning models in biology will continuously be limited by experimental data, and plants pose a particular challenge in this context. Their metabolism is highly compartmentalized comprising, compared to animal cells, additional compartments like the vacuole, plastid, and cell wall. Applying combined experimental protocols for subcellular fractionation and *omics* analysis can provide high-throughput data, which is suitable for quantitative data integration on a large-scale (Fürtauer et al., 2019). Finally, plant metabolism is highly dynamic due to diurnal or seasonal changes of the environment, which might be analyzed by differential equation (DE) models as discussed within the following section.

## Differential Equation Models for Quantitative Analysis of Biochemical Network Dynamics

Mathematical models of plant metabolism are frequently based on systems of DEs. For example, dynamics of metabolite concentrations are mathematically described in such models by the sum of synthesizing and interconverting/degrading enzyme reactions. Typically, time is considered to be the only independent variable, and, thus, ordinary differential equations (ODEs) are applied for simulating biochemical networks (Andrews and Arkin, 2006). If two or more independent variables are considered, e.g., time and space, partial differential equations (PDEs) are applied.

To briefly illustrate the suitability of (ordinary) differential equations for dynamic modeling of metabolism, consider an arbitrary enzyme catalyzed two-substrate reaction (Eq. 1):

(1) $$A+B\overset{k}{\to }C$$

Here, two substrate molecules A and B react to form a product C with the rate constant *k*. Changes of substrate and product concentrations within a time period *Δt* (infinitesimally written as *dt*) are captured by the corresponding ODEs (Eq. 2):

(2) $$-\frac{d\left[A\right]}{dt}=-\frac{d\left[B\right]}{dt}=\frac{d\left[C\right]}{dt}=k\left[A\right]\left[B\right]=f\left(A,B,C\right)$$

The right side of the ODEs can be summarized by metabolic functions ***f***(A, B, C) comprising all (kinetic) terms, which contribute to changes in concentration of substrate and product molecules. While in this arbitrary example metabolic functions only comprise one kinetic term, the composition of such functions in metabolic systems are much more complex due to various enzyme reactions, which contribute to synthesis, degradation, or transport of metabolites. Also, while kinetics in Eq. 2 are described as constantly proportional to substrate concentrations without regulatory impact, enzyme catalyzed reactions typically follow kinetics with saturation, inhibition, and activation. Systems of DEs mathematically amalgamate different kinetic laws with dynamic substrate, product, and effector concentrations, which enable quantitative simulation of metabolism. Further, DEs enable different types of kinetic modeling focusing on dynamic (time-series) data or steady-state approaches (Rohwer, 2012). However, for simulation of kinetic DE models within physiologically relevant boundaries, sets of kinetic parameters and metabolite concentrations need to be quantified. As a consequence, due to experimental limitations, the applicability of (O)DE-based models is frequently limited to relatively small networks and narrow time frames, in which the model can explain or reliably predict experimental data. Nevertheless, DEs constitute a very important approach for modeling of metabolic networks because of the inbuilt consideration of substrate and product concentrations on metabolic functions, i.e., a changing substrate concentration has a direct effect on its own metabolic function (Nägele, 2014).

In a metabolic DE model, each differential equation describes dynamics of one metabolite. Thus, modeling a metabolic network results in a system of DEs, which needs to be solved, i.e., numerically integrated, within biochemical and physiological boundaries. Numerical integration of (O)DEs can be performed computationally using platforms like Copasi (Hoops et al., 2006; Kent et al., 2012), Python (van Rossum, 1995), or R (R Core Team, 2021). Boundaries for solving ODEs arise from experiments and typically comprise information about SD/error of kinetic parameters, protein, or metabolite concentration. Within the process of parameter estimation, kinetic parameters are determined to reflect experimental data on metabolite or protein concentrations with a minimized error (Moles et al., 2003). Hence, the more precise experimental quantification of such parameters and concentration is the less ambiguous are solutions of equation systems. Yet, previous findings also indicated that parameter measurements must be highly precise and complete in order to minimize “sloppiness” in parameter sensitivities and to usefully constrain model predictions (Gutenkunst et al., 2007). Based on their findings, the authors suggest to focus rather on validation of model predictions than on model parameters. Although uncertainties about model structure, parameters or kinetic laws can hardly be excluded from future modeling approaches due to their nested architecture (Schaber et al., 2009), an iterative workflow consisting of model development, simulation, and validation by quantitative experiments will refine and advance model output and predictive power (Babtie and Stumpf, 2017). Such modeling approaches have revealed detailed insights into molecular processes comprising, e.g., regulatory motifs of moonlighting proteins (Krantz and Klipp, 2020), temperature compensation in reaction networks (Ruoff et al., 2007), or mechanisms regulating diurnal starch dynamics (Pokhilko et al., 2014).

## Future Perspective and Conclusion

**Figure 3:**
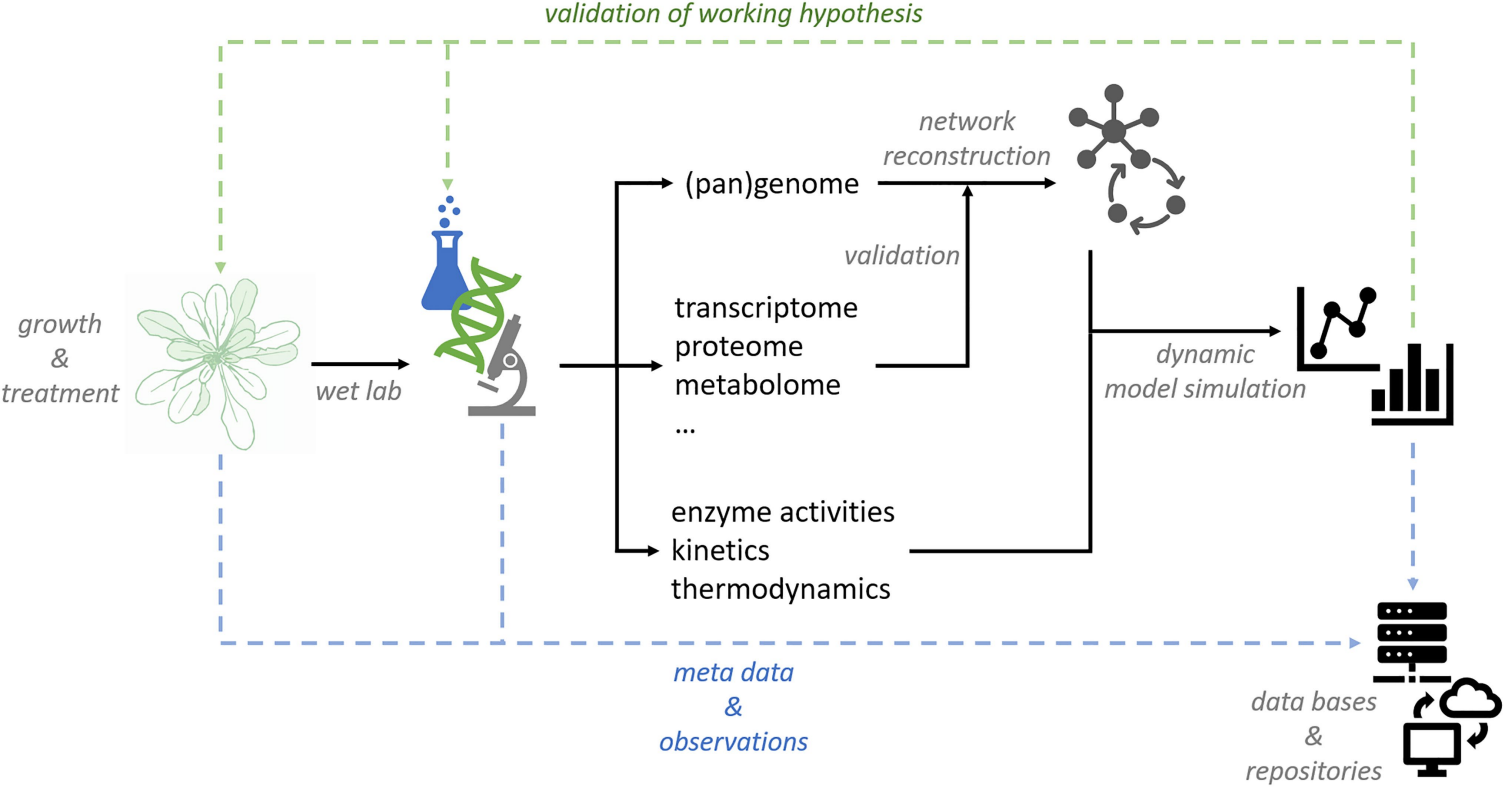
Conceptual workflow for data management and modeling in plant sciences.

Due to tremendous progress in experimental high-throughput analysis, well conceptualized research data management systems are becoming essential for sustainable data storage and labeling. Simultaneously, quantitative analysis of plant metabolism on large scale will support combination and comparison of complex data originating from different labs or research platforms. Bioinformatics and -mathematics play a central role both in data management and modeling due to their capability to manage, integrate and analyze multidimensional data sets. In combination with dynamic mathematical models, network structures elucidated by (pan)genome-based network reconstruction will yield mechanistic insight into regulation of plant metabolism (Figure 3).

Finally, beyond its role as a tool for understanding and analyzing experimental data on plant metabolism, mathematical modeling also enables the comparison to structure and regulation of other complex systems in nature and engineering, which will support and accelerate the identification of underlying universal principles of biochemical network organization, regulation, and architecture.

## Author Contributions

MK and DZ are first authors and contributed equally to this manuscript. TN conceived and wrote the manuscript. All authors contributed to the article and approved the submitted version.

## Conflict of Interest

The authors declare that the research was conducted in the absence of any commercial or financial relationships that could be construed as a potential conflict of interest.

## Publisher’s Note

All claims expressed in this article are solely those of the authors and do not necessarily represent those of their affiliated organizations, or those of the publisher, the editors and the reviewers. Any product that may be evaluated in this article, or claim that may be made by its manufacturer, is not guaranteed or endorsed by the publisher.

## Footnotes

^1^ https://pubmed.ncbi.nlm.nih.gov/

^2^ https://www.re3data.org/

^3^ https://dataverse.org/
